# *In vitro* antibacterial activity of extracts and purified fractions from the marine sponge *Suberites* aff. *latus* (Demospongiae, Suberitida, Suberitidae) from Ica, Peru

**DOI:** 10.17843/rpmesp.2025.424.15003

**Published:** 2025-12-13

**Authors:** Erin Villanueva-Coronado, Juan C. Francia-Quiroz, Christian Polo, Oscar Reategui, Báslavi Cóndor-Luján, Aldo G. Indacochea, Maria J. Pons

**Affiliations:** 1 Marine Biology Program, Faculty of Veterinary and Biological Sciences, Universidad Científica del Sur, Lima, Peru. Universidad Científica del Sur Marine Biology Program Faculty of Veterinary and Biological Sciences Universidad Científica del Sur Lima Peru; 2 Research Group on Characterization, Transformation, and Sustainability of Peru’s Natural Resources (CTS Group), Universidad Científica del Sur, Lima, Peru. Universidad Científica del Sur Research Group on Characterization Transformation, and Sustainability of Peru’s Natural Resources (CTS Group) Universidad Científica del Sur Lima Peru; 3 Department of Zoology, Faculty of Biological Sciences, Universidad Nacional Mayor de San Marcos, Lima, Peru. Universidad Nacional Mayor de San Marcos Department of Zoology Faculty of Biological Sciences Universidad Nacional Mayor de San Marcos Lima Peru; 4 Emerging and Re-emerging Infectious Diseases Group, Universidad Científica del Sur, Lima, Peru. Universidad Científica del Sur Emerging and Re-emerging Infectious Diseases Group Universidad Científica del Sur Lima Peru

**Keywords:** Minimum Inhibitory Concentration, Microdilution, Chemical Screening, Paracas, Porifera, Bioactivity

## Abstract

Marine sponges constitute a recognized source of metabolites with potential antibacterial activity. In the present study, the antibacterial activity of the marine sponge *Suberites* aff. *latus* from Ica, Peru, was evaluated. Lyophilized sponge samples were extracted using dichloromethane:methanol (1:1), and fractionated by solid-phase extraction into seven fractions (F1-F7), ranging from aqueous to organic polarity. The minimum inhibitory concentration was determined against Gram-positive/negative bacterial strains from the ATCC (American Type Culture Collection) collection. The results showed that the highest activity was observed in fraction F6 of specimen 01J against *Klebsiella pneumoniae* and *Pseudomonas aeruginosa*, as well as in fraction F3 of specimen 02J against *K. pneumoniae*. Qualitative chemical analysis revealed the presence of alkaloids and saponins in the samples. These findings highlight the potential of *Suberites* aff. *latus* from the Peruvian coast as a promising source of antibacterial compounds.

## INTRODUCTION

The misuse of antimicrobial agents has led to a significant increase in antimicrobial resistance (AMR), driving the search for drugs in new sources such as sponges ^1^. Marine sponges (phylum Porifera) are an abundant source of marine natural products (MNP), with 9,231 compounds reported, including pharmaceutical properties ^2^. Sponges of the genus *Suberites* (Demospongiae, Suberitida, Suberitidae) are widely distributed globally and frequently inhabit temperate waters. In this genus, the presence of ASABF-type antimicrobial peptides (Antheraea samia antibacterial factor, defensin-type) has been documented in *Suberites domuncula*^4^; alkaloids (Nakijinamines) isolated from *Suberites sp.* with cytotoxic and antibacterial activity ^5^; and extracts of *Suberites* luna showed bioactivity against pathogens such *as Staphylococcus epidermidis* and *Enterococcus faecalis*^6^.

The Peruvian coast has great potential for the bioprospecting of compounds from marine sponges ^7^. Therefore, the objective of this study was to evaluate the in vitro antibacterial activity of crude extracts and purified fractions of *Suberites* aff. *latus*, a sponge distributed along the northern (05°), central (12°), and southern (14°) coasts of Peru ^3^.

**KEY MESSAGES**

Motivation for the study. Marine sponges constitute a source of metabolites with antibacterial potential that will be explored in the absence of new antimicrobials.

Main findings. Variable antibacterial activity was observed in the four samples of *Suberites* aff. *latus*, despite belonging to the same species. The potential of fractions F3 (H₂O/MeOH: 1/3) and F6 (MeOH/DCM: 1/1) stands out with lower Minimum Inhibitory Concentration (MIC) values.

Public health implications. This study suggests that *Suberites* aff. *latus* from Ica (Peru) is a promising source of antibacterial compounds, although further research is required to identify the active metabolites and understand the factors influencing their bioactivity.

## THE STUDY

### Sampling of biological material

**Figure 1:**
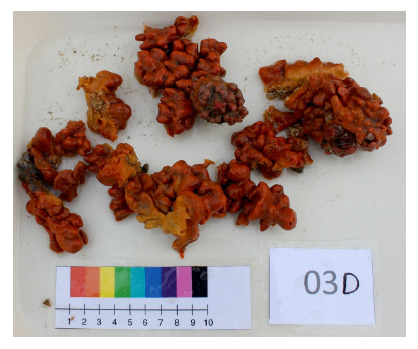
Ex situ specimen of Suberites aff. latus, collected on Sangayán Island, Paracas.

The collection and preservation of *Suberites* aff. *latus* (Figure 1) were carried out in Paracas, located in Ica (Peru), an area characterized by the influence of the Peru Current and, in turn, the San Juan de Marcona upwelling (Ica). This region features cold waters, low oxygen concentration, high productivity, and nutrient concentration ^8^.

Credits: Juan C. Francia-Quiroz

**Figure 2:**
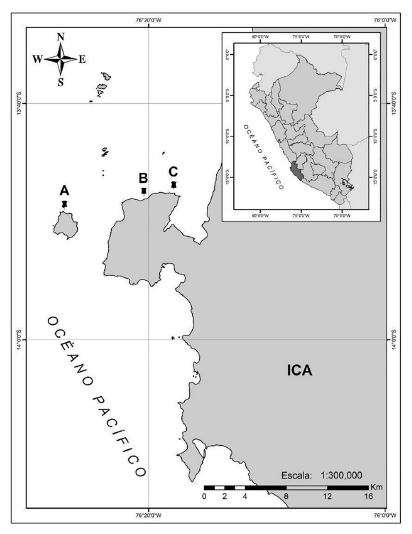
Collection sites of Suberites aff. latus in Paracas, Ica, Peru. (A) San-gayán Island. (B) Talpo. (C) Punta Ripio

Samples were collected in January 2020 via semi-autonomous diving at three sites in Paracas, specifically in Talpo, Punta Ripio, and Sangayán Island (Figure 2, supplementary material table s1), following the methodology proposed by Cóndor-Luján and Francia-Quiroz ^9^. Once epibionts were removed, samples were transferred to plastic bags and transported to the Universidad Científica del Sur (Lima) under cold chain. In the Molecular Genetics Laboratory, they were stored in freezers at -35 °C and subsequently at -80 °C.

Collection was conducted under research authorization from the Ministry of Production (R.D. N° 00396-2020-PRODUCE/DGPCHDI) and the study was approved by the Institutional Ethics Committee for Research with Animals and Biodiversity of the Universidad Científica del Sur (N° 109-CIEI-AB-CIENTÍFICA-2021).

### Lyophilization

Lyophilization was performed following the methodology proposed by Cóndor-Luján and Francia-Quiroz ^9^. Samples were fragmented with tweezers to remove remains of associated organisms (endobionts), impurities, and adhered sediments. Wet and dry weights were recorded after lyophilization (supplementary material table s2). Subsequently, samples were lyophilized at -105 °C and 0.013 mbar using a LABCONCO FreeZone lyophilizer, manually pulverized with a mortar, and stored at -35 °C until extraction.

### Extraction and fractionation

For extraction and obtaining purified fractions, the previously proposed methodology was followed with some modifications ^9^^,^^10^. Maceration was performed with the organic solvents dichloromethane/methanol (1:1; 3×100 mL) and 4 g of the pulverized lyophilisate. It was homogenized in a sonicator (Branson 2510) at room temperature for 10 min at 60 rpm.

It was filtered by gravity using Whatman No. 2 filter paper, and solvents were removed under vacuum using a rotary evaporator (Selecta RS 3001-V). The obtained solid residue was fractionated using the solid-phase extraction (SPE) technique. Seven solvent mixtures with decreasing polarity were used (60 mL each): (1) H2O; (2) H2O/MeOH (1:1); (3) H2O/MeOH (1:3); (4) MeOH; (5) MeOH/DCM (3:1); (6) MeOH/DCM (1:1); (7) DCM. Fractions and the crude extract were resuspended at different concentrations (10-100 mg/mL) in 10% dimethyl sulfoxide.

### Qualitative chemical analysis

Screening was performed according to the methodology proposed by Lock ^11^ and Dominguez ^12^, with some modifications. Extraction was carried out using 100 mg of each lyophilized sponge sample and 40 mL of methanol (MeOH). It was placed in the sonicator for 10 min. The methanolic extract was filtered using Whatman No. 2 paper. The resulting solution was divided into two parts: A1 (5 mL) and A2 (35 mL). Solution A1 was placed in a water bath at 100 °C until complete evaporation of the solvent. This solid residue was stored for the detection of alkaloids and saponins.

Alkaloids were evaluated after the addition of 1% HCl using Dragendorff’s reagent (positive: orange-brown precipitate) and Mayer’s reagent (positive: white to cream precipitate). Saponins were detected by the formation of stable foam after resuspension and vigorous shaking of A1 in 2 mL of hot distilled water (3-5 min).

Methanolic solution A2 was used to detect ketones, coumarins, and phenols. Ketones were identified using Legal’s reagent (positive: dark red coloration). Phenols were evaluated with FeCl3 (positive: intense blue or green coloration). Finally, coumarins were detected with Baljet’s reagent (positive: light to dark red coloration).

### Antibacterial activity tests

*Microorganisms*

Gram-negative bacterial strains of Escherichia coli (ATCC 25922), Pseudomonas aeruginosa (ATCC 27853), and Klebsiella pneumoniae (BAA 1706) were used, as well as Gram-positive strains of Staphylococcus aureus (ATCC 25923) and Bacillus subtilis (ATCC 6633). Bacteria were resuspended in a test tube with saline solution (0.5X) at a concentration of 0.5 McFarland. Subsequently, a 1/100 dilution for Gram-positive bacteria and a 1/1000 dilution for Gram-negative bacteria in ultrapure water was performed ^6^.

*Minimum inhibitory concentration (MIC)*

The antibacterial activity of crude extracts and purified fractions was evaluated using the in vitro microdilution technique to determine the MIC, as previously described ^6^. For the experiment, 96-well plates were used. In each well, an initial volume of 50 µL of Mueller-Hinton broth was added, followed by the addition of 50 µL of the corresponding crude extract or fraction. Serial microdilutions were then performed, and finally, 10 µL of bacterial suspension were introduced into the plates. Final concentrations of the extracts and fractions evaluated ranged from 0.39 to 50 mg/mL, and the test was performed in triplicate. For the growth control (positive control), 50 µL of Mueller-Hinton broth and 10 µL of bacteria were used. For the sterility control (negative control), 50 µL of Mueller-Hinton broth was used. Plates were incubated at a temperature of 37 °C for a period of 18 to 24 hours, and results were evaluated based on the presence of visible turbidity. The MIC was identified as the lowest concentration of the tested extracts that effectively prevents visible bacterial growth.

## FINDINGS

Four crude extracts were obtained from the total samples. Among these, the yield of the crude extracts was 17.60% (01J), 16.76% (02J), 25.34% (02A), and 20.87% (03D) (supplementary material table s2). All four samples of *Suberites* aff. *latus* showed antibacterial activity against at least one of the analyzed strains. Table 1 presents a summary of the minimum inhibitory concentration (MIC) results for both crude extracts and purified fractions. The crude extract of sample 01J showed antibacterial activity against *E. coli, B. subtilis* (MIC = 6.25 mg/mL), and *P. aeruginosa* (MIC = 3.13 mg/mL). The highest inhibitory activity (MIC = 1.25 mg/mL) was detected in F6 (MeOH/DCM 1:1) of sample 01J against *K. pneumoniae* and *P. aeruginosa*, and in F3 (H2O/MeOH 1:1) of sample 02A against *P. aeruginosa*.

**Table 1:** Minimum inhibitory concentration (MIC) (mg/mL) of crude extracts and fractions of *Suberites* aff. *latus* collected in Paracas.

Sample code		Gram-negative			Gram-positive		Control	
		***K. pneumoniae* BAA1706**	***E. coli* ATCC 25922**	***P. aeruginosa* ATCC 27853**	***B. subtilis* ATCC 6633**	***S. aureus* ATCC 25923**	Growth control	Sterility control
01J	CE	>12.50	6.25	3.13	6.25	>12.50	+	-
F1	>12.50	>12.50	>12.50	>12.50	>12.50	+	-
F2	12.50	6.25	6.25	12.50	12.50	+	-
F3	12.50	6.25	6.25	12.50	12.50	+	-
F4	3.13	3.13	3.13	6.25	1.56	+	-
F5	>12.5	>12.50	>12.50	>12.50	>12.50	+	-
F6	1.25	2.50	1.25	2.50	2.50	+	-
F7	6.25	6.25	6.25	6.25	6.25	+	-
02A	CE	>12.50	>12.50	>12.50	>12.50	>12,50	+	-
	F1	>12.50	>12.50	12.50	>12.50	>12.50	+	-
	F2	12.50	12.50	12.50	>12.50	>12.50	+	-
	F3	2.50	2.50	1.25	5	5	+	-
	F4	6.25	3.13	6.25	6.25	12.50	+	-
	F5	>12.50	>12.50	>12.50	>12.50	>12.50	+	-
	F6	2.50	2.50	5	5	5	+	-
	F7	5	5	5	>5	>5	+	-
02J	CE	>12.50	>12.50	>12.50	>12.50	>12.50	+	-
	F1	>12.50	>12.50	>12.50	>12.50	>12.50	+	-
	F2	>12.50	>12.50	>12.50	>12.50	>12.50	+	-
	F3	12.50	12.50	12.50	12.50	12.50	+	-
	F4	12.50	6.25	6.25	6.25	3.13	+	-
	F5	>12.50	>12.50	>12.50	>12.50	>12.50	+	-
	F6	5	5	5	5	5	+	-
	F7	2.50	2.50	2.50	2.50	5	+	-
03D	CE	>12.50	>12.50	>12.50	>12.50	>12.50	+	-
	F1	>50	>50	>50	>50	>50	+	-
	F2	>50	>50	>50	>50	>50	+	-
	F3	>50	>50	>50	>50	>50	+	-
	F4	>25	>25	>25	>25	>25	+	-
	F5	>12.50	>12.50	12.50	>12.50	12.50	+	-
	F6	>5	>5	>5	>5	>5	+	-
	F7	>5	>5	>5	>5	>5	+	-

(+): presence, (-): absence. Highlighted MIC values indicate antibacterial activity.

CE: crude extract; ; F1: Mili-Q H₂O; F2: Mili-Q H₂O/MeOH (1:1); F3: Mili-Q H₂O/MeOH (1:3); F4: MeOH; F5: MeOH/DCM (3:1); F6: MeOH/DCM (1:1).

According to the qualitative chemical analysis, the four analyzed samples presented alkaloids and saponins. Phenolic compounds were found in samples 03D and 01J. Coumarins were detected in samples 01J and 02J. Finally, ketones were only found in sample 02J (Table 2).

**Table 2:** Qualitative chemical analysis of the four samples of *Suberites* aff. *latus.*

Sample code	Chemical screening					
	Alkaloids		Phenolic compounds	Saponins	Coumarins	Ketones
	Dragendorff	Mayer				
03D	+	+	+	+	-	-
01J	+	+	+	+	+	-
02A	+	-	-	+	-	-
02J	+	-	-	+	+	+

(+): presence, (-): absence.

## DISCUSSION

The results of this study indicate broad-spectrum activity against Gram-positive and Gram-negative pathogens, coinciding with previous studies on extracts of sponges of the genus *Suberites*^6^^,^^13^. However, efficacy is lower compared to purified molecules; for example, the compound Nakijinamine A from *Suberites sp.* collected in Okinawa showed an MIC of only 0.016 mg/mL against *Staphylococcus aureus* and *B. subtilis*^5^, while in this study, the MIC was 1.56 and 6.25 mg/mL, respectively. This difference is explained because the activity of the crude extract may be masked by the high concentration of inactive compounds or salts present ^14^. Furthermore, the extraction method and solvent polarity are crucial factors influencing the type and diversity of metabolites obtained, resulting in variable antibacterial activities ^15^.

Fractions F3 (polar, H2O/MeOH 1:3) and F6 (non-polar, MeOH/DCM 1:1) demonstrated superior inhibitory activity (MIC up to 1.25 mg/mL) compared to crude extracts. This increase in efficacy is expected when using the Solid-Phase Extraction technique, whose purpose is selective separation by polarity and the consequent increase in efficacy ^16^. The activity observed in these fractions indicates the simultaneous presence of different secondary metabolites of hydrophilic (F3) and lipophilic (F6) nature. This diversity is characteristic of marine sponges, whose active metabolites span chemical classes with a broad antimicrobial spectrum such as terpenoids, sterols, and alkaloids ^14^.

Based on qualitative chemical analysis, our results reported the presence of alkaloids and saponins. Alkaloids have potential as antibacterials, as they inhibit nucleic acid synthesis and cell division or alter bacterial homeostasis ^17^.

Variation in antibacterial activity and chemical groups was observed among the four collected samples, possibly due to physiological differences, energy reserves, health, size, reproductive state of the sponge, and environmental conditions at the collection site ^18^.

Samples collected in Talpo and Punta Ripio showed greater antibacterial activity despite being closer to areas with anthropogenic impact and effluents from the Pisco River ^8^. A greater abundance of bacterioplankton could have activated a defense mechanism. Proteins similar to the perforins of *Suberites domuncula* showed protection against bacterial epibiosis ^13^^),^ and these proteins could serve as a defense mechanism for the species under study.

The sample collected at Sangayán Island was found in a kelp forest. Competition with macroalgae affects the distribution of sponges in shallow waters ^18^^)^ and influences the production of secondary metabolites ^19^. The activity of photosynthetic symbiont microorganisms in sponges can be affected by the reduction of irradiance in kelp forests ^18^, which reduces the energy reserves of the sponges and affects their ability to produce secondary metabolites. Furthermore, microorganisms associated with sponges produce secondary metabolites with broad-spectrum bioactivity ^20^. Environmental conditions can affect the sponge microbiome, causing variable bioactivity in sponges of the same species ^20^^).^

Regarding limitations, the evaluation of bacterial growth was performed through visible turbidity observation, which lacks the quantitative precision offered by spectrometry. Likewise, phenotypic and genotypic techniques were not included, which limits the ability to distinguish between a bacteriostatic and bactericidal effect, as well as to identify molecular mechanisms of action. This methodological limitation prevents specifying whether the active compounds originate from the sponge, its microbial symbionts, or the interaction between them. Therefore, it is suggested that future studies include phenotypic and molecular techniques to determine the biosynthetic origin of the metabolites and evaluate the effect of environmental conditions on the metabolome of sponges and their associated microorganisms ^13^.

In conclusion, variable antibacterial activity was observed among the four samples of *Suberites* aff. *latus*, despite belonging to the same species. The potential of fractions F3 (H₂O / MeOH: 1/3) and F6 (MeOH / DCM: 1/1) with lower MIC values stands out. This research highlights the potential of *Suberites* aff. *latus* from Ica (Peru) as a source of bioactive compounds against the studied bacteria. However, future studies are required to identify the active compounds with antibacterial activity and determine their origin, as well as studies on the biotic and abiotic factors that affect the demonstrated bioactivity.
