# Supplementary material for: In vitro antibacterial activity of extracts and purified fractions from the marine sponge Suberites aff. latus (Demospongiae, Suberitida, Suberitidae) from Ica, Peru
Source: Rev Peru Med Exp Salud Publica. 2025 Dec 13;42(4):424–9. doi: 10.17843/rpmesp.2025.424.15003 (PMC12879985; doi:10.17843/rpmesp.2025.424.15003)
Supplement: Supplementary material. — Available in the electronic version of the RPMESP. [file rpmesp-42-04-15003-s001.docx]

Material suplementario:

Tabla S1. Datos de las localidades de recolección de *Suberites* aff. *latus* de Ica, Perú

| **Código de muestra** | **Localidad** | **Coordenadas geográficas** | **Profundidad(m)** | **Temperatura (°C)** | **Fecha de colecta** |
| --- | --- | --- | --- | --- | --- |
|  |  |  |  |  |  |
| 01J | Talpo | 13°48'01.8''S 76°20'24.7''O | 4.10 | 16 | 21/01/2020 |
| 02J | Talpo | 13°48'01.8''S 76°20'24.7''O | 2.50 | 16-17 | 21/01/2020 |
| 02A | Punta Ripio | 13°47'31.1''S 76°17'50.8''O | 1.50 | * | 21/01/2020 |
| 03D | Isla Sangayan | 13°49'4.0''S 76°27'08.0''O | 4.50 | 17 | 22/01/2020 |

* No disponible

Tabla S2. Datos de la liofilización y extracción de *Suberites* aff. *latus*

| **Código de muestra** | **Peso húmedo (g)** | **Peso seco (g)** | **Residuo sólido (g)** | **Rendimiento de extracción (%)** |
| --- | --- | --- | --- | --- |
| 01J | 198 | 50 | 1.15 | 17.60 |
| 02J | 327 | 106 | 1.07 | 16.76 |
| 02A | 58 | 13 | 1.42 | 25.34 |
| 03D | 146 | 40 | 1.29 | 20.87 |
