# Supplementary material for: In vitro antibacterial activity of extracts and purified fractions from the marine sponge Suberites aff. latus (Demospongiae, Suberitida, Suberitidae) from Ica, Peru
Source: Rev Peru Med Exp Salud Publica. 2025 Dec 13;42(4):424–9. doi: 10.17843/rpmesp.2025.424.15003 (PMC12879985; doi:10.17843/rpmesp.2025.424.15003)
Supplement: Translation

# Translation: Actividad antibacteriana *in vitro* de extractos y fracciones purificadas de la esponja marina *Suberites* aff. *Latus* (Demospongiae, Suberitida, Suberitidae) de Ica, Perú

**Authors:** Erin Villanueva-Coronado, Juan C. Francia-Quiroz, Christian Polo, Oscar Reategui, Báslavi Cóndor-Luján, Aldo G. Indacochea, Maria J. Pons

**Affiliations:** 1 Carrera de Biología Marina, Facultad de Ciencias Veterinarias y Biológicas, Universidad Científica del Sur, Lima, Perú.; 2 Grupo de Investigación, Caracterización, Transformación y Sustentabilidad de los Recursos Naturales del Perú (CTS Group), Universidad Científica del Sur, Lima, Perú.; 3 Departamento de Zoología, Facultad de Ciencias Biológicas, Universidad Nacional Mayor de San Marcos, Lima, Perú.; 4 Grupo de Enfermedades Infecciosas (re) emergentes, Universidad Científica del Sur, Lima, Perú.

**Keywords:** Concentración Mínima Inhibitoria, Microdilución, Análisis Químico, Paracas, Porifera, Bioactividad

## Abstract

Las esponjas marinas constituyen una fuente reconocida de metabolitos con potencial actividad antibacteriana. En el presente estudio se evaluó la actividad antibacteriana de la esponja marina *Suberites* aff*. latus* de Ica, Perú. Las muestras de esponja liofilizadas se extrajeron utilizando diclorometano:metanol (1:1), y se fraccionaron mediante extracción en fase sólida en siete fracciones (F1-F7), que iban de la polaridad acuosa a la orgánica. Se determinó la concentración mínima inhibitoria frente a cepas bacterianas Grampositivas/negativas de colección ATCC (American Type Culture Collection). Los resultados mostraron que la mayor actividad fue observada en la fracción F6 del espécimen 01J frente a *Klebsiella pneumoniae* y *Pseudomonas aeruginosa*, así como en la fracción F3 del espécimen 02J frente a *K. pneumoniae*. El análisis químico cualitativo reveló la presencia de alcaloides y saponinas en las muestras. Estos hallazgos ponen de relieve el potencial de *Suberites* aff. *latus* de la costa peruana como fuente prometedora de compuestos antibacterianos.

## INTRODUCCIÓN

El uso indebido de agentes antimicrobianos ha provocado un aumento significativo de la resistencia a los antimicrobianos (AMR) y ello ha impulsado la búsqueda de fármacos en nuevas fuentes como las esponjas ^1^. Las esponjas marinas (filo Porifera) son una fuente abundante de productos naturales marinos (MNP), con 9231 compuestos reportados incluyendo propiedades farmacéuticas ^2^. Las esponjas del género *Suberites* (Demospongiae, Suberitida, Suberitidae) se encuentran ampliamente distribuidas en el mundo y frecuentemente habitan aguas templadas. En este género, se ha documentado la presencia de péptidos antimicrobianos de tipo ASABF (*Antheraea samia* antibacterial factor, tipo defensina) en *Suberites domuncula*^4^, alcaloides (Nakijinaminas) aislados de *Suberites* sp. con actividad citotóxica y antibacteriana ^5^ y extractos de *Suberites luna* mostraron bioactividad contra patógenos como *Staphylococcus epidermidis* y *Enterococcus faecalis*^6^.

La costa peruana tiene gran potencial para la bioprospección de compuestos de esponjas marinas ^7^. Por ello, el objetivo de este estudio fue evaluar la actividad antibacteriana *in vitro* de extractos crudos y fracciones purificadas de *Suberites* aff*. latus*, una esponja con distribución en la costa norte (05°), central (12°) y sur (14°) del Perú ^3^.

**MENSAJE CLAVE**

Motivación para realizar el estudio. Las esponjas marinas constituyen una fuente de metabolitos con potencial antibacteriano que se explorará en ausencia de nuevos antimicrobianos.

Principales hallazgos. Se observó una actividad antibacteriana variable en las cuatro muestras de *Suberites* aff. *latus*, a pesar de pertenecer a la misma especie. Se destaca el potencial de las fracciones F3 (H₂O/MeOH: 1/3) y F6 (MeOH/DCM: 1/1) con valores más bajos de Concentración Mínima Inhibitoria (CMI).

Implicancias en salud pública. Este estudio sugiere que *Suberites* aff. *latus* de Ica (Perú) es una fuente prometedora de compuestos antibacterianos, aunque se requiere más investigación para identificar los metabolitos activos y comprender los factores que influyen en su bioactividad.

## EL ESTUDIO

### Muestreo de material biológico

La recolección y preservación de *Suberites* aff*. latus* (figura 1) se realizó en Paracas, ubicada en Ica (Perú), una zona caracterizada por recibir la influencia de la Corriente del Perú y a su vez, el afloramiento de San Juan de Marcona (Ica). Esta región presenta aguas frías, baja concentración de oxígeno, alta productividad y concentración de nutrientes ^8^.

**Figura 1:** Muestra ex situ de *Suberites* aff. *latus*, recolectada en la isla Sanga-yán, Paracas.

Créditos: Juan C. Francia-Quiroz

Las muestras se recolectaron en enero de 2020 mediante buceo semiautónomo en tres lugares de Paracas, específicamente en Talpo, Punta Ripio y la isla de Sangayán (figura 2, material suplementario tabla s1), siguiendo la metodología propuesta por Cóndor-Luján y Francia-Quiroz ^9^. Una vez retirados los epibiontes, las muestras se transfirieron a bolsas plásticas y se transportaron a la Universidad Científica del Sur (Lima) en cadena de frío. En el Laboratorio de Genética molecular, se almacenaron en congeladores a -35 °C y posteriormente a -80 °C.

**Figura 2:** Lugares de colecta de *Suberites* aff. *latus* en Paracas, Ica, Perú. (A) Isla Sangayán. (B) Talpo. (C) Punta Ripio.

La colecta se efectuó bajo la autorización de investigación del Ministerio de Producción (R.D. N° 00396-2020-PRODUCE/DGPCHDI) y el estudio fue aprobado por el Comité Institucional de Ética en Investigación con Animales y Biodiversidad de la Universidad Científica del Sur (N° 109-CIEI-AB-CIENTÍFICA-2021).

### Liofilización

La liofilización se realizó siguiendo la metodología propuesta por Cóndor-Luján y Francia-Quiroz ^9^. Las muestras se fragmentaron con pinzas para eliminar los restos de organismos asociados (endobiontes), impurezas y sedimentos adheridos. Se registraron los pesos húmedo y seco tras la liofilización (material suplementario tabla s2). Posteriormente, las muestras se liofilizaron a -105 °C y 0,013 mbar utilizando el liofilizador LABCONCO FreeZone, se pulverizaron manualmente con mortero y se almacenaron a -35 °C hasta su extracción.

### Extracción y fraccionamiento

Para la extracción y obtención de fracciones purificadas, se siguió la metodología propuesta previamente con algunas modificaciones ^9^^,^^10^. La maceración se realizó con los disolventes orgánicos diclorometano/metanol (1:1; 3×100 mL) y 4 g del liofilizado pulverizado. Se homogeneizó en un sonicador (Branson 2510) a temperatura ambiente durante 10 min y 60 rpm.

Se filtró por gravedad utilizando papel de filtro Whatman N°. 2, y los disolventes se eliminaron al vacío utilizando el evaporador rotatorio (Selecta RS 3001-V). El residuo sólido obtenido se fraccionó utilizando la técnica de extracción en fase sólida (SPE). Se utilizaron 60 mL de siete mezclas de disolventes con polaridad decreciente: (1) H2O; (2) H2O/MeOH (1:1); (3) H2O/MeOH (1:3); (4) MeOH; (5) MeOH/DCM (3:1); (6) MeOH/DCM (1:1); (7) DCM. Las fracciones y el extracto crudo se resuspendieron a diferentes concentraciones (10-100 mg/mL) en dimetilsulfóxido al 10%.

### Análisis químico cualitativo

El cribado se realizó según la metodología propuesta por Lock ^11^ y Dominguez ^12^, con algunas modificaciones. La extracción se realizó utilizando 100 mg de cada muestra de esponja liofilizada y 40 mL de metanol (MeOH). Se colocó en el sonicador durante 10 min. El extracto metanólico se filtró utilizando papel Whatman Nº 2. La solución resultante se dividió en dos partes: A1 (5 mL) y A2 (35 mL). La solución A1 se colocó en un baño de agua a 100 °C hasta la completa evaporación del solvente. Este residuo sólido se almacenó para la detección de alcaloides y saponinas.

Los alcaloides se evaluaron tras la adición de HCl al 1% mediante los reactivos de Dragendorff (positivo: precipitado anaranjado-marrón) y de Mayer (positivo: precipitado blanco a crema). Las saponinas se detectaron por la formación de espuma estable tras la resuspensión y agitación vigorosa de A1 en 2 mL de agua destilada caliente (3-5 min).

La solución metanólica A2 se utilizó para detectar cetonas, cumarinas y fenoles. Las cetonas se identificaron mediante el reactivo Legal (positivo: coloración rojo oscuro). Los fenoles se evaluaron con FeCl_3_ (positivo: coloración azul o verde intenso). Finalmente, las cumarinas se detectaron con el reactivo Baljet (positivo: coloración rojo claro a oscuro).

### Pruebas de actividad antibacteriana

*Microorganismos*

Se utilizaron cepas bacterianas gramnegativas de *Escherichia coli* (ATCC 25922), *Pseudomonas aeruginosa* (ATCC 27853) y *Klebsiella pneumoniae* (BAA 1706), así como grampositivas de *Staphylococcus aureus* (ATCC 25923) y *Bacillus subtilis* (ATCC 6633). Las bacterias se resuspendieron en un tubo de ensayo con solución salina (0,5X) a una concentración de 0,5 McFarland. Posteriormente, se realizó una dilución 1/100 para bacterias grampositivas y 1/1000 para bacterias gramnegativas en agua ultrapura ^6^.

*Concentración mínima inhibitoria (CMI)*

La actividad antibacteriana de los extractos crudos y las fracciones purificadas se evaluó mediante la técnica de microdilución *in vitro* para determinar la CMI, como se describió previamente ^6^. Para el experimento, se utilizaron placas de 96 pocillos. En cada pocillo, se añadió un volumen inicial de 50 µL de caldo Muller Hinton, seguido de la adición de 50 µL del extracto crudo o fracción correspondiente. A continuación, se realizaron microdiluciones seriadas y, finalmente, se introdujeron 10 µL de suspensión bacteriana en las placas. Las concentraciones finales de los extractos y fracciones evaluadas oscilaron entre 0,39 a 50 mg/mL, y se realizó por triplicado. Para el control de crecimiento (control positivo), se utilizaron 50 µL de caldo Muller Hinton y 10 µL de bacterias. Para el control de esterilidad (control negativo), se utilizaron 50 µL de caldo Muller Hinton. Las placas se incubaron a una temperatura de 37 °C durante un período de 18 a 24 horas, y los resultados se evaluaron en función de la presencia de turbidez visible. La CMI se identificó como la concentración más baja de los extractos probados que previene eficazmente el crecimiento bacteriano visible.

## HALLAZGOS

Se obtuvieron cuatro extractos crudos a partir del total de muestras. Entre estos, el rendimiento de los extractos crudos fue de 17,60% (01J), 16,76% (02J), 25,34% (02A) y 20,87% (03D) (material suplementario tabla s2). Las cuatro muestras de *Suberites* aff. *latus* mostraron actividad antibacteriana contra al menos una de las cepas analizadas. La Tabla 1 presenta un resumen de los resultados de la concentración mínima inhibitoria (CMI) tanto para los extractos crudos como para las fracciones purificadas. El extracto crudo de la muestra 01J mostró actividad antibacteriana contra *E. coli, B. subtilis* (CMI = 6,25 mg/mL) y *P. aeruginosa* (CMI = 3,13 mg/mL). La mayor actividad inhibitoria (CMI=1,25 mg/mL) se detectó en el F6 (MeOH/DCM 1:1) de la muestra 01J contra *K. pneumoniae* y *P. aeruginosa* y en el F3 (H2O/MeOH 1:1) de la muestra 02A contra *P. aeruginosa.*

**Tabla 1:** Concentración mínima inhibitoria (CMI) (mg/mL) de extractos crudos y fracciones de *Suberites* aff. *latus* recolectadas en Paracas.

Código de muestra		Gramnegativo			Grampositivo		Control	
		***K. pneumoniae* BAA1706**	***E. coli* ATCC 25922**	***P. aeruginosa* ATCC 27853**	***B. subtilis* ATCC 6633**	***S. aureus* ATCC 25923**	Control de crecimiento	Control de esterilidad
01J	CE	>12,50	6,25	3,13	6,25	>12,50	+	-
	F1	>12,50	>12,50	>12,50	>12,50	>12,50	+	-
	F2	12,50	6,25	6,25	12,50	12,50	+	-
	F3	12,50	6,25	6,25	12,50	12,50	+	-
	F4	3,13	3,13	3,13	6,25	1,56	+	-
	F5	>12,5	>12,50	>12,50	>12,50	>12,50	+	-
	F6	1,25	2,50	1,25	2,50	2,50	+	-
	F7	6,25	6,25	6,25	6,25	6,25	+	-
02A	CE	>12,50	>12,50	>12,50	>12,50	>12,50	+	-
	F1	>12,50	>12,50	12,50	>12,50	>12,50	+	-
	F2	12,50	12,50	12,50	>12,50	>12,50	+	-
	F3	2,50	2,50	1,25	5	5	+	-
	F4	6,25	3,13	6,25	6,25	12,50	+	-
	F5	>12,50	>12,50	>12,50	>12,50	>12,50	+	-
	F6	2,50	2,50	5	5	5	+	-
	F7	5	5	5	>5	>5	+	-
02J	CE	>12,50	>12,50	>12,50	>12,50	>12,50	+	-
	F1	>12,50	>12,50	>12,50	>12,50	>12,50	+	-
	F2	>12,50	>12,50	>12,50	>12,50	>12,50	+	-
	F3	12,50	12,50	12,50	12,50	12,50	+	-
	F4	12,50	6,25	6,25	6,25	3,13	+	-
	F5	>12,50	>12,50	>12,50	>12,50	>12,50	+	-
	F6	5	5	5	5	5	+	-
	F7	2,50	2,50	2,50	2,50	5	+	-
03D	CE	>12,50	>12,50	>12,50	>12,50	>12,50	+	-
	F1	>50	>50	>50	>50	>50	+	-
	F2	>50	>50	>50	>50	>50	+	-
	F3	>50	>50	>50	>50	>50	+	-
	F4	>25	>25	>25	>25	>25	+	-
	F5	>12,50	>12,50	12,50	>12,50	12,50	+	-
	F6	>5	>5	>5	>5	>5	+	-
	F7	>5	>5	>5	>5	>5	+	-

(+) presencia, (-) ausencia. Los valores MIC resaltados indican actividad antibacteriana.

CE: extracto crudo; F1: Mili-Q H₂O; F2: Mili-Q H₂O/MeOH (1:1); F3: Mili-Q H₂O/MeOH (1:3); F4: MeOH; F5: MeOH/DCM (3:1); F6: MeOH/DCM (1:1).

Según el análisis químico cualitativo, las cuatro muestras analizadas presentaron alcaloides y saponinas. Se encontraron compuestos fenólicos en las muestras 03D y 01J. Se detectaron cumarinas en las muestras 01J y 02J. Finalmente, solo se encontraron cetonas en la muestra 02J (tabla 2).

**Tabla 2:** Análisis químico cualitativo de las cuatro muestras de *Suberites* aff. *latus.*

Código de muestra	Cribado químico					
	Alcaloides		Compuestos fenólicos	Saponinas	Cumarinas	Cetonas
	Dragendorff	Mayer				
03D	+	+	+	+	-	-
01J	+	+	+	+	+	-
02A	+	-	-	+	-	-
02J	+	-	-	+	+	+

(+) presencia, (-) ausencia

## DISCUSIÓN

Los resultados de este estudio indican una actividad de amplio espectro contra patógenos grampositivos y gramnegativos, coincidiendo con estudios previos sobre extractos de esponjas del género *Suberites*^6^^,^^13^. No obstante, la eficacia es menor en comparación con moléculas purificadas, por ejemplo, el compuesto Nakijinamina A de *Suberites* sp. colectado en Okinawa mostró CMI de solo 0,016 mg/mL contra *Staphylococcus aureus* y *B. subtilis*^5^, mientras que en este estudio el CMI fue de 1,56 y 6,25 mg/mL, respectivamente. Esta diferencia se explica porque la actividad del extracto crudo puede estar enmascarada por la alta concentración de compuestos inactivos o sales presentes ^14^. Además, el método de extracción y la polaridad del solvente son factores cruciales que influyen en el tipo y la diversidad de metabolitos obtenidos, resultando en actividades antibacterianas variables ^15^.

Las fracciones F3 (polar, H_2_O/MeOH 1:3) y F6 (apolar, MeOH/DCM 1:1) demostraron una actividad inhibitoria superior (CMI hasta 1,25 mg/mL) a la de los extractos crudos. Este incremento en eficacia es esperado al usar la técnica de Extracción en Fase Sólida, cuyo propósito es la separación selectiva por polaridad y el consecuente aumento de eficacia ^16^. La actividad observada en estas fracciones indica la presencia de diferentes metabolitos secundarios de naturaleza hidrofílica (F3) y lipofílica (F6), simultáneamente. Esta diversidad es característica de las esponjas marinas, cuyos metabolitos activos abarcan clases químicas de amplio espectro antimicrobiano como terpenoides, esteroles y alcaloides ^14^.

Con base en el análisis químico cualitativo, nuestros resultados reportaron la presencia de alcaloides y saponinas. Los alcaloides tienen potencial como antibacterianos, ya que inhiben la síntesis de ácidos nucleicos y la división celular o alteran la homeostasis bacteriana ^17^.

Se observó variación en la actividad antibacteriana y los grupos químicos entre las cuatro muestras recolectadas, posiblemente debido a diferencias fisiológicas, reserva energética, salud, tamaño, estado reproductivo de la esponja y condiciones ambientales del sitio de recolección ^18^.

Las muestras recolectadas en Talpo y Punta Ripio mostraron mayor actividad antibacteriana a pesar de estar más cerca de áreas con impacto antropogénico y de los efluentes del río Pisco ^8^. Una mayor abundancia de bacterioplancton podría haber activado un mecanismo de defensa. Proteínas similares a las perforinas de *Suberites domuncula* mostraron protección contra la epibiosis bacteriana ^13^, y estas proteínas podrían servir como mecanismo de defensa para la especie en estudio.

La muestra recolectada en la isla Sangayán se encontró en un bosque de algas. La competencia con macroalgas afecta la distribución de las esponjas en aguas someras ^18^ e influye en la producción de metabolitos secundarios ^19^. La actividad de los microorganismos simbiontes fotosintéticos de las esponjas puede verse afectada por la reducción de la irradiancia en los bosques de algas marinas ^18^, lo que reduce la reserva energética de las esponjas y afecta su capacidad para producir metabolitos secundarios. Además, los microorganismos asociados a las esponjas producen metabolitos secundarios con una bioactividad de amplio espectro ^20^. Las condiciones ambientales pueden afectar el microbioma de las esponjas, causando una bioactividad variable en esponjas de la misma especie ^20^.

En cuanto a las limitaciones, la evaluación del crecimiento bacteriano se realizó mediante la observación de la turbidez visible, el cual carece de la precisión cuantitativa que ofrece la espectrometría. Asimismo, no se incluyó técnicas fenotípicas y genotípicas lo cual limita la capacidad de distinguir entre un efecto bacteriostático y bactericida, así como identificar los mecanismos moleculares de acción. Esta limitación metodológica impide especificar si los compuestos activos provienen de la esponja, de sus simbiontes microbianos o de la interacción entre ellos. Por lo tanto, se sugiere que estudios futuros incluyan técnicas fenotípicas y moleculares para determinar el origen biosintético de los metabolitos y evaluar el efecto de las condiciones ambientales en el metaboloma de las esponjas y sus microorganismos asociados ^13^.

En conclusión, se observó una actividad antibacteriana variable entre las cuatro muestras de *Suberites* aff*. latus*, a pesar de pertenecer a la misma especie. Se destaca el potencial de las fracciones F3 (H₂O / MeOH: 1/3) y F6 (MeOH / DCM: 1/1) con valores de CMI más bajos. Esta investigación destaca el potencial de *Suberites* aff. *latus* de Ica (Perú) como fuente de compuestos bioactivos contra las bacterias estudiadas. Sin embargo, se requieren estudios futuros para identificar los compuestos activos con actividad antibacteriana y determinar su origen, así como estudios sobre los factores bióticos y abióticos que afectan la bioactividad demostrada.
